# Agreement between diagnostic imaging methods for the evaluation of lymphadenopathies in HIV-infected/AIDS patients

**DOI:** 10.1590/0100-3984.2017.0176

**Published:** 2019

**Authors:** Francisco Carlos da Silva, Gabriel Antonio Nascentes, Antonio Carlos Oliveira Meneses, Dalmo Correia Filho

**Affiliations:** 1 Universidade Federal do Triângulo Mineiro (UFTM), Uberaba, MG, Brazil.

**Keywords:** HIV, Acquired immunodeficiency syndrome, Lymphadenopathy, Ultrasonography, Tomography, X-ray computed, Magnetic resonance imaging, HIV, Síndrome da imunodeficiência adquirida, Linfadenopatia, Ultrassonografia, Tomografia computadorizada, Ressonância magnética

## Abstract

**Objective:**

To assess the percent agreement between diagnostic imaging modalities for the evaluation of lymphadenopathies in HIV-infected/AIDS patients.

**Materials and Methods:**

This was an open, comparative, prospective study of diagnostic imaging methods for lymphadenopathy evaluation. We evaluated 30 patients (19 men and 11 women). All underwent ultrasound and computed tomography (CT). Twenty of the patients also underwent magnetic resonance imaging (MRI). We determined the percent agreement between two examiners using the various imaging methods to evaluate lymphadenopathies.

**Results:**

CT had the highest percent agreement, at 93.3%, with a kappa coefficient of 0.85, corresponding to 28 of the 30 examinations. When we compared the percent agreement between the two examiners and between CT and ultrasound, examiner 1 had an observed rate of 80.0%, with a kappa of 0.49, corresponding to 24 of the 30 examinations, whereas examiner 2 had a rate of 70.0%, with a kappa of 0.31, corresponding to 21 of the 30 examinations. Between MRI and CT, the percent agreement for examiner 1 was 50.0%, with a kappa of −0.18, corresponding to 10 of the 20 examinations, whereas that for examiner 2 was 85.0%, with a kappa of 0.69, corresponding to 17 of the 20 examinations. For MRI and ultrasound, examiner 1 had a percent agreement of 70.0%, with a kappa of 0.20, corresponding to 14 of the 20 examinations, and examiner 2 had a percent agreement of 75.0%, with a kappa of 0.38, corresponding to 15 of the 20 examinations.

**Conclusion:**

This study indicates that intermethod agreement is highly dependent on the way in which the research is conducted, rather than on the level of experience of the examiner.

## Abstract (pt)

**Objetivo:**

Avaliar a porcentagem de concordância entre métodos de diagnóstico por imagem na avaliação de linfonodomegalias em pacientes HIV(+)/AIDS.

**Materiais e Métodos:**

Foi realizado estudo aberto, comparativo e prospectivo dos métodos de diagnóstico por imagem na avaliação de linfonodomegalias. Foram avaliados 30 pacientes (19 homens e 11 mulheres). Todos foram submetidos a ultrassonografia e tomografia computadorizada (TC) e 20 também foram submetidos a ressonância magnética (RM). Foi determinado o grau de concordância entre dois examinadores usando-se métodos de diagnóstico por imagem.

**Resultados:**

A TC obteve o maior grau de concordância, com taxa de 28/30 (93,3%) e coeficiente kappa de 0,85. Quando comparado o grau de concordância para linfonodomegalias entre dois examinadores e entre TC e ultrassonografia, o examinador 1 teve taxa de 24/30 (80,0%) e kappa de 0,49, e o examinador 2 teve taxa de 21/30 (70,0%) e kappa de 0,31. Entre RM e TC, o examinador 1 teve taxa de 10/20 (50,0%) e kappa de -0,18, e o examinador 2 teve taxa de 17/20 (85,0%) e kappa de 0,69. Entre RM e ultrassonografia, o examinador 1 teve taxa de 14/20 (70,0%) e kappa de 0,20, e o examinador 2 teve taxa de 15/20 (75,0%) e kappa de 0,38.

**Conclusão:**

Este estudo indica que a concordância entre métodos depende fortemente da forma como a pesquisa é conduzida e não da capacidade do examinador.

## INTRODUCTION

Acquired immunodeficiency syndrome (AIDS) is caused by infection with HIV, a retrovirus that exhibits tropism for cells of the immune system and central nervous system, affecting CD4^+^ T lymphocytes in particular^(^1^,^^2^^)^. HIV, which belongs to the genus *Lentivirus*, has a molecular structure comprising 15 proteins encoded by two RNA molecules^(^3^)^.

Worldwide, there are nearly 37.6 million people living with HIV infection, and 2.1 million new cases were reported in 2015; a significant number of those occurred in African countries. In Latin America, a total of 2 million cases have been reported; more than a third of those occurred in Brazil^(^4^)^. Recent data suggest that the HIV epidemic will end by 2030, given that reductions in the numbers of new cases exceed 50% in some countries^(^5^)^. In Brazil, there is currently a clear trend toward an increase in the incidence of AIDS among young people, particularly among women 13-19 years of age, which has reduced the male:female ratio, the number of AIDS cases per 100,000 population being 2.0 among males and 1.6 among females^(^4^)^.

Superficial and deep lymphadenopathies are among the main clinical manifestations of early- and late-stage AIDS; the latter often featuring inflammatory, infectious, or neoplastic comorbidities^(^6^,^^7^^)^. Physiologically, lymph nodes have a diameter of 1.0-1.5 cm, feature an oval shape, have predominantly central vascularization, and are located in surface or cavitary chains^(^6^)^.

In HIV-infected/AIDS patients, lymphadenopathies appear early in the infectious process, as a component of disease progression, and can occur in response to opportunistic infectious agents (e.g., bacteria, fungi, and viruses) or malignant degeneration (e.g., lymphoma and sarcoma)^(^8^)^. The most common causes of lymphadenopathy in HIV-infected/AIDS patients are diseases related to infection with bacteria, mycobacteria (e.g., tuberculosis), fungi (e.g., histoplasmosis, cryptococcosis, and paracoccidioidomycosis), or viruses (e.g., cytomegalovirus infection and herpes virus infection), as well as those related to colonization by parasites (e.g., toxoplasmosis and leishmaniasis). Such lymphadenopathies are often generalized and minimally painful, mainly affecting the cervical and retroperitoneal lymph nodes. Patients with tuberculosis nearly always originate from endemic areas and exhibit some degree of pulmonary impairment^(^9^)^.

Lymphoproliferative disorders also comprise part of the spectrum of lymphadenopathies in HIV-infected/AIDS patients. Among such disorders, the most common histological types are B-cell lymphomas, Hodgkin lymphoma, and Burkitt lymphoma^(^10^-^12^)^.

Imaging modalities are important tools in the diagnosis of lymphadenopathies. Several imaging methods can be used to characterize the condition as inflammatory (infectious or not) or related to malignancy (primary or metastatic).

Because of its ease of use, accessibility, and low cost, ultrasound examination is indicated for the evaluation of peripheral lymphadenopathies. However, computed tomography (CT) and magnetic resonance imaging (MRI) are the best methods for evaluating cavitary lymphadenopathies^(^13^-^16^)^.

CT can clearly characterize lymph nodes, distinguish them from neighboring structures, and suggest an inflammatory, infectious, or malignant etiology. The disadvantage of CT is the use of iodinated contrast, which is contraindicated in many patients (e.g., those with diabetes, kidney failure, or allergies).

The efficacy of MRI is similar and, in some cases, superior to that of CT. Therefore, MRI can be used to characterize the extent of lesions and distinguish lymph nodes from lesions, as well as to determine the number, volume, and appearance of lesions^(^7^)^.

The objective of the present study was to evaluate the level of agreement between diagnostic imaging methods (ultrasound, CT, and MRI) for the evaluation of lymphadenopathies. Specifically, we compared the three methods in terms of their efficacy in evaluating superficial and deep lymphadenopathies in HIV-infected/AIDS patients.

## MATERIALS AND METHODS

This was a prospective, cross-sectional, open, comparative study of the diagnostic imaging methods available for the evaluation of lymphadenopathies at the Universidade Federal do Triângulo Mineiro (UFTM)-Federal University of Triângulo Mineiro-between February 2012 and September 2013. We also compared the results with those of the gold standard methods (histopathological analysis and culture). The study was approved by the UFTM Research Ethics Committee (Protocol No. 2327).

We recruited HIV-infected/AIDS patients with a fever of unclear etiology, abdominal pain, wasting syndrome, superficial or cavitary inflammatory lymphadenopathies, or acute abdominal inflammation who were treated in the Department of Infectious and Parasitic Diseases, Clinical Ward, or Emergency Room of the UFTM Clinical Hospital during the period under study. We also included patients seen at the Infectious and Parasitic Diseases Outpatient Clinic of the UFTM Clinical Hospital.

This final sample comprised 30 patients (19 males and 11 females). The mean age was 42 years (range, 20-61 years). These demographic characteristics are consistent with those of many published studies on this topic^(^17^-^19^)^. All participating patients gave written informed consent.

Diagnostic imaging examinations (ultrasound, CT, and MRI) were performed by two different examiners, both of whom were blinded to the serological status of the patients. Each examiner issued reports independently (without any knowledge of the reports issued by the other examiner). All imaging examinations were performed in the Imaging Department of the UFTM Clinical Hospital.

### Ultrasound evaluation

All of the patients underwent ultrasound evaluation. We employed an Accuvix V10 ultrasound system (Samsung Medison, Seoul, South Korea), using a 5-12 MHz linear probe to study surface lymphadenopathies and a 3-5 MHz convex probe to study cavitary lymphadenopathies. For the purposes of this study, the following examination protocol was established: analysis of anatomical features (size, shape, echotexture, topography, number, presence of calcifications, central necrosis, distribution of vascularization, adherence to deep layers, and presence of hemorrhage); and analysis of characteristics suggestive of malignancy (e.g., anteroposterior diameter greater than the longitudinal diameter, invasion of surrounding tissues, and loss of corticomedullary differentiation). Color Doppler analysis was also used.

### CT evaluation

All of the patients underwent CT evaluation. In all CT examinations, we used a 64-channel multislice spiral CT scanner (Aquilion; Toshiba Corporation, Tokyo, Japan) and particular attention was given to lymphadenopathies detected previously by ultrasound. All patients except those with a history of hypersensitivity to iodinated agents received intravenous contrast via peripheral venipuncture in an upper limb. Patients were exposed to a minimal radiation dose because all slices were obtained simultaneously in a fraction of 10-15 s. During the CT scans, in addition to the aspects evaluated via ultrasound, we evaluated the presence or absence of contrast uptake and peripheral enhancement.

### MRI evaluation

For economic reasons, only 20 patients underwent MRI scans for comparison with the ultrasound and CT images. All of those patients underwent MRI examination in a 1.5 T scanner (Avanto; Siemens AG, Berlin, Germany). The MRI scans allowed better evaluation of cavitary lymphadenopathies and included additional elements, such as multiplanar reformatting, as well as more detailed analyses of anatomical, inflammatory, and neoplastic aspects. Gadolinium contrast was used.

The descriptive CT and MRI findings were compared with the ultrasound data in terms of the levels of agreement and disagreement regarding the parameters related to lymphadenopathy (size, number, shape, location, necrosis, calcifications, distinction from surrounding tissues, vascularization, corticomedullary differentiation, adherence to deep layers, and hepatosplenomegaly).

### Statistical analysis

The statistical analysis of categorical data was conducted using appropriate tests. Possible associations between risk factors and the presence of lymphadenopathy were assessed by using the chi-square test with Yates correction, and kappa coefficients were calculated with 95% confidence intervals. The kappa coefficient was used in order to assess agreement between the diagnostic tests, as well as between the examiners. The level of agreement was based on the indices suggested by Landis and Koch, as follows: kappa ≤ 0 = none; 0.01-0.40 = weak; 0.41-0.60 = discreet; 0.61-0.80 = moderate; 0.81-0.99 = substantial; and 1.00 = perfect. Statistical analyses were performed using Statistica software, version 10.0 (Statsoft Inc., Tulsa, OK, USA).

## RESULTS

Between February 2012 and September 2013, 81 patients with a confirmed diagnosis of HIV infection/AIDS with fever and lymphadenopathies underwent ultrasound evaluation. The mean age of those patients was 42 years (range, 20-61 years). Of those 81 patients, 30 (11 females and 19 males) were selected to undergo ultrasound and CT scans, 20 of those 30 being selected to undergo MRI as well. The remaining 51 patients were excluded for the following reasons: hospital discharge before the examinations; refusal of admission to the hospital; death before completion of examinations (especially histopathology); or specific constraints regarding each imaging method (e.g., metallic prostheses or artificial pacemakers, for MRI, and allergy to iodine, for CT).

Sixteen patients (53%) underwent fine-needle aspiration biopsy. Among those 16 patients, the results were inconclusive in 14 (87.5%) and lymphoid hyperplasia was detected in two (12.5%). Thirty patients underwent lymph node excision, and the subsequent histopathological study revealed necrosis in one. The following histopathological diagnoses were made: lymphoid hyperplasia, in six patients (20%); tuberculosis, in six (20%); nonspecific chronic inflammation, in four (14%); histoplasmosis, in three (10%); paracoccidioidomycosis, in three (10%); acute suppurative inflammation, in two (7%); Hodgkin lymphoma, in one (3%); B-cell lymphoma, in one (3%); toxoplasmosis, in one (3%); and leishmaniasis, in one (3%). The lymphadenopathies were distributed as follows: in the cervical region, in 25 cases (83%); in the abdominal region, in 18 (60%); in the thoracic region, in three (10%); in the inguinal region, in three (10%); in the axillary region, in three (10%); in the retroperitoneal region, in one (0.3%); in the periaortic region, in one (0.3%); and in the peripancreatic region, in one (0.3%).

**Figure 1:**
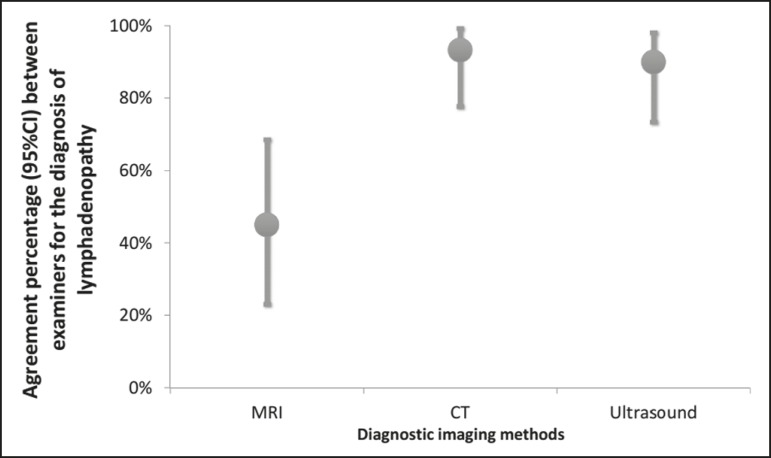
Interexaminer agreement in the diagnosis of lymphadenopathies with various imaging methods.

We evaluated the percent agreement (95% CI) between the two examiners in the diagnosis of lymphadenopathies for each of the three diagnostic imaging methods (Figure 1). The highest percent agreement was observed for the CT scans-93.3% (95% CI: 77.9% to 99.2%), with a kappa coefficient of 0.85 (95% CI: 0.65 to 1.00), corresponding to 28 of the 30 examinations.

**Figure 2:**
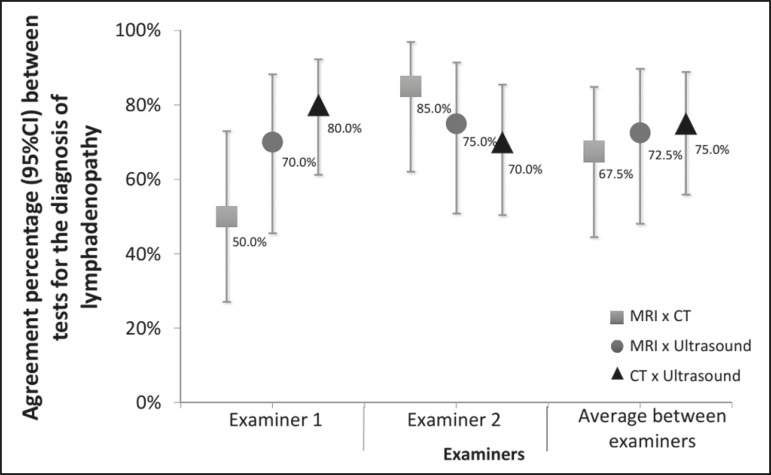
Percent agreement between imaging methods for the diagnosis of lymphadenopathies, according to both examiners.

The percent agreement between CT and ultrasound, between MRI and CT, and between MRI and ultrasound were calculated for both examiners. As can be seen in Figure 2, the agreement between CT and ultrasound was stronger for examiner 1 than for examiner 2, whereas the inverse was true for the agreement between MRI and CT.

**Figure 3:**
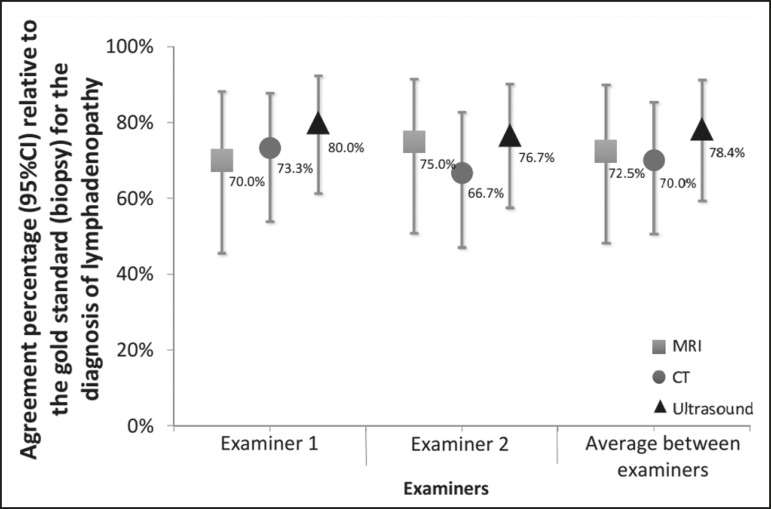
Percent agreement between imaging tests and the gold standard methods, according to both examiners.

In our analysis of the percent agreement between the examination results and those of the gold standard methods (histopathology and culture), we observed no significant interexaminer differences. The ultrasound examinations showed the highest percent agreement with the gold standard methods (Figure 3).

## DISCUSSION

In the HIV-infected/AIDS patients evaluated in the present study, lymphadenopathies were attributed to various diseases, including lymphoid hyperplasia, suppurative inflammatory processes, nonspecific chronic inflammation, infectious processes (e.g., tuberculosis, paracoccidioidomycosis, and histoplasmosis), and parasitic diseases (e.g., toxoplasmosis and leishmaniasis). In addition, some patients presented with neoplastic diseases such as Hodgkin lymphoma and B-cell lymphoma.

Our findings confirm the supposition that lymphadenopathies are common manifestations in HIV-infected/AIDS patients. According to our initial hypothesis, a standardized diagnostic imaging analysis regarding parameters indicative of morphological, functional, inflammatory, and malignant aspects would increase the level of agreement between the methods, thus reducing dependence on the examiner. However, our results did not confirm our initial hypothesis. Despite the use of sophisticated diagnostic imaging methods such as CT and MRI, the observed dependence on the examiner remained significant.

We proposed this study to address the difficulty in diagnosing lymphadenopathy in HIV-infected/AIDS patients with fever and wasting syndrome, as well as the lack of studies demonstrating a correlation between radiological findings and etiology. Our ultimate intention was to contribute to the implementation of a standardized protocol for the diagnostic evaluation of lymphadenopathies in HIV-infected/AIDS patients at the UFTM. We hope that the results of our analyses will also facilitate future evaluations of HIV-infected/AIDS patients.

Our initial hypothesis suggested that better interexaminer agreement could reduce the dependence on the examiner during the selection of a diagnostic imaging method. However, the fallacy of this hypothesis became evident because, for many of the aspects under study, ultrasound was more accurate than were CT and MRI.

Lymphadenopathy in HIV-infected/AIDS patients continues to be a controversial topic, requiring future research in the field of diagnostic imaging, as well as in other areas. We hope that the present study will raise questions regarding the best diagnostic imaging approach for the evaluation of lymphadenopathies. For example, would an ultrasound examination be sufficient for the evaluation of superficial lymphadenopathies? Would only CT or MRI be sufficient the evaluation of for cavitary lymphadenopathies? Further studies concerning lymphadenopathies in HIV-infected/AIDS patients should address such questions, while considering factors such as the time required to conduct each method, contrast use, cost, and level of experience of the examiner.

We found that the aspects of time, resolution, location, and examiner experience had stronger impacts on the diagnosis than did the imaging method employed. Ease of use is a significant advantage of ultrasound examination, especially for superficial lymphadenopathies. Specifically, an ultrasound examination can be completed rapidly, and a subsequent guided biopsy can be performed within minutes. That is not true of MRI, which requires 40-50 min for the examination; accordingly, scheduling may not allow a patient to undergo MRI after an ultrasound evaluation. However, the poor ability of ultrasound to detect thoracic and retroperitoneal lymphadenopathies, which are easily visualized on CT and MRI, represents a major limitation of the former. Finally, our findings demonstrate that intermethod agreement often depends not on examiner experience but on the manner in which the research is conducted.

Our study has some limitations, including factors such as refusal of admission to the hospital, death before completion of examinations (especially histopathology), and specific constraints regarding each imaging method. The highest percent agreement regarding overall diagnostic imaging, for both examiners, was between ultrasound and CT. Regarding the percent agreement for the diagnosis of lymphadenopathies, the best agreement was between CT and ultrasound for examiner 1 and between MRI and CT for examiner 2. In addition, ultrasound yielded the highest percent agreement with the gold standard, for both examiners. On the basis of this isolated analysis of imaging tests for the diagnosis of lymphadenopathies, we cannot make inferences regarding etiological agents, regardless of the imaging method employed.

In conclusion, the effects that time, resolution, location, and examiner experience have on diagnosis are stronger than are those of the imaging modality. The ease of ultrasound examination constitutes a significant advantage, particularly for superficial lymphadenopathies.
